# Activation of GPR4 by Acidosis Increases Endothelial Cell Adhesion through the cAMP/Epac Pathway

**DOI:** 10.1371/journal.pone.0027586

**Published:** 2011-11-16

**Authors:** Aishe Chen, Lixue Dong, Nancy R. Leffler, Adam S. Asch, Owen N. Witte, Li V. Yang

**Affiliations:** 1 Department of Internal Medicine, Brody School of Medicine, East Carolina University, Greenville, North Carolina, United States of America; 2 Department of Anatomy and Cell Biology, Brody School of Medicine, East Carolina University, Greenville, North Carolina, United States of America; 3 UNC Lineberger Comprehensive Cancer Center, Chapel Hill, North Carolina, United States of America; 4 Howard Hughes Medical Institute, University of California Los Angeles, Los Angeles, California, United States of America; Universidade de Sao Paulo, Brazil

## Abstract

Endothelium-leukocyte interaction is critical for inflammatory responses. Whereas the tissue microenvironments are often acidic at inflammatory sites, the mechanisms by which cells respond to acidosis are not well understood. Using molecular, cellular and biochemical approaches, we demonstrate that activation of GPR4, a proton-sensing G protein-coupled receptor, by isocapnic acidosis increases the adhesiveness of human umbilical vein endothelial cells (HUVECs) that express GPR4 endogenously. Acidosis in combination with GPR4 overexpression further augments HUVEC adhesion with U937 monocytes. In contrast, overexpression of a G protein signaling-defective DRY motif mutant (R115A) of GPR4 does not elicit any increase of HUVEC adhesion, indicating the requirement of G protein signaling. Downregulation of GPR4 expression by RNA interference reduces the acidosis-induced HUVEC adhesion. To delineate downstream pathways, we show that inhibition of adenylate cyclase by inhibitors, 2′,5′-dideoxyadenosine (DDA) or SQ 22536, attenuates acidosis/GPR4-induced HUVEC adhesion. Consistently, treatment with a cAMP analog or a G_i_ signaling inhibitor increases HUVEC adhesiveness, suggesting a role of the G_s_/cAMP signaling in this process. We further show that the cAMP downstream effector Epac is important for acidosis/GPR4-induced cell adhesion. Moreover, activation of GPR4 by acidosis increases the expression of vascular adhesion molecules E-selectin, VCAM-1 and ICAM-1, which are functionally involved in acidosis/GPR4-mediated HUVEC adhesion. Similarly, hypercapnic acidosis can also activate GPR4 to stimulate HUVEC adhesion molecule expression and adhesiveness. These results suggest that acidosis/GPR4 signaling regulates endothelial cell adhesion mainly through the G_s_/cAMP/Epac pathway and may play a role in the inflammatory response of vascular endothelial cells.

## Introduction

Local or systemic acidosis is associated with a variety of pathological conditions such as inflammation, ischemia, tumor, diabetic ketoacidosis, and lung and renal diseases due to defective blood flow, hypoxia, and glycolytic metabolism [1], [2], [3], [4], [5], [6]. For instance, interstitial pH in ischemic organs often decreases to 7.0 - 6.0 and sometimes even below 6.0 [5], [7], [8]. There are two major types of acidosis: isocapnic acidosis, such as metabolic acidosis caused by excessive metabolic acids, and hypercapnic acidosis, such as respiratory acidosis caused by carbon dioxide accumulation [9], [10], [11]. Acidosis has profound effects on blood vessels, immune cells, inflammatory responses, and tissue injury [4], [8], [12], [13], [14], [15], but the molecular mechanisms by which acidosis regulates vascular function, endothelium-leukocyte interaction and inflammation are not well known.

The GPR4 family of proton-sensing G protein-coupled receptors (GPCRs) has recently been identified as novel pH sensors [15], [16], [17], [18], [19], [20], [21]. GPR4, originally cloned as an orphan GPCR, is expressed in a wide range of tissues such as the lung, kidney, heart, and liver [22], [23], [24]. GPR4 is highly conserved during evolution, with more than 90% amino acid sequence homology among mammalian orthologs and more than 70% homology between human and zebrafish orthologs. However, the biological function of GPR4 is not clearly defined. GPR4 was previously reported as a receptor for sphingosylphosphorylcholine (SPC) and lysophosphatidylcholine (LPC) [25], but this observation has not always been confirmed [20], [26], [27] and the original publication has been withdrawn [25]. Several studies indicated that GPR4 mediates the SPC-induced endothelial tube formation, LPC-induced impairment of endothelial barrier function, and LPC-induced vascular cell adhesion molecule-1 (VCAM-1) expression [28], [29], [30]. Since the ligand-receptor relationship between SPC, LPC and GPR4 is not validated, it is unclear whether GPR4 directly or indirectly mediates the biological effects of SPC and LPC.

More recent studies from several research groups demonstrated that GPR4 predominantly functions as a proton sensor activated by extracellular acidic pH [15], [16], [17], [20]. Protonation of several extracellular histidine residues of GPR4 is important for the receptor activation [16], [17]. GPR4, together with OGR1, TDAG8 and G2A, comprise a novel proton-sensing GPCR family [15], [16], [17], [18], [19], [20], [21]. We have recently shown that activation of GPR4 by acidosis stimulates the G_s_/cyclic adenosine monophosphate (cAMP) signaling in endothelial cells and regulates microvessel growth [15]. GPR4-null neonatal mice exhibit a partially penetrant phenotype of spontaneous hemorrhaging with small blood vessel defects [15]. Recent analysis of GPR4-null mice has revealed that GPR4 is involved in acid-base homeostasis in the kidney [31]. These data suggest that GPR4 is a functional proton sensor in cells.

GPR4 can be stimulated by extracellular acidic pH to transduce downstream signals through G_s_/cAMP, G_q_/phospholipase C (PLC), and G_13_/Rho pathways [15], [16], [17], [20]. cAMP is a ubiquitous second messenger that controls a wide range of cellular processes mainly through the downstream effector protein kinase A (PKA). More recently, Epac (exchange protein directly activated by cAMP) has been identified as a new effector of cAMP and is involved in many important cellular processes including cell adhesion [32], [33]. The cAMP/Epac/Rap1 signaling pathway has been reported to regulate a number of PKA-independent processes, such as β2-adrenergic receptor-mediated ovarian carcinoma cell adhesion to fibronectin [34], monocyte adhesion and chemotaxis [35], and adenosine receptor-stimulated activation of ERK1/2 in HUVEC cells [36].

Here we have identified a novel function of GPR4 in response to acidosis. Activation of GPR4 by acidic pH increases endothelial cell adhesion with leukocytes and this biological effect is mediated through the cAMP/Epac pathway. Acidosis/GPR4 signaling also up-regulates the expression of several adhesion molecules in endothelial cells. These findings suggest that the acidosis/GPR4 signaling may represent a new pathway in vascular inflammation responding to acidic microenvironments in many pathological conditions.

## Materials and Methods

### Reagents and chemicals

2′,5′-dideoxyadenosine (DDA), SQ 22536, 8-bromoadenosine 3′,5′-cyclic monophosphate (8-Br-cAMP), 8-(*p*-chlorophenylthio)-2′-*O*-methyladenosine-3′,5′-cyclic monophosphate (8-CPT-2Me-cAMP), H-89, and pertussis toxin (PTX) were purchased from Calbiochem/EMD4Biosciences (La Jolla, CA). 3-isobutyl-1-methylxanthine (IBMX), 4-(2-hydroxyethyl)-1-piperazineethanesulfonic acid (HEPES), *N*-(2-hydroxyethyl)-piperazine-*N′*-3-propanesulfonic acid (EPPS), 2-(4-morpholino)-ethanesulfonic acid (MES), and protease inhibitor cocktail were from Sigma-Aldrich (St Louis, MO) and Fisher Scientific (Fair Lawn, NJ). Anti-c-myc mouse monoclonal antibody (clone 9E10) was purchased from Roche Diagnostics (Indianapolis, IN). E-selectin (clone BBIG-E4), VCAM-1 (clone BBIG-V1), inter-cellular adhesion molecule-1 (ICAM-1) (clone BBIG-I1) and P-selectin (clone 9E1) monoclonal antibodies were from R&D Systems (Minneapolis, MN).

### Plasmid constructs

The open reading frame of human GPR4 (GenBank accession number NM 005282) was amplified from human cDNAs by polymerase chain reaction (PCR) with the high-fidelity pfu-turbo DNA polymerase (Stratagene, La Jolla, CA). The PCR primer pair was: sense strand 5′- ATAAGAATGCGGCCGCACCATGGGCAACCACACGTG -3′ and antisense strand 5′- ATAAGAATGCGGCCGCTCATTGTGCTGGCGGCAGCAT -3′. A NotI site was introduced at each end of the PCR fragment for molecular cloning and the Kozak consensus sequence was added before the translation start codon ATG for efficient translation. The amplified PCR fragment was digested with NotI and cloned into the retroviral expression vector MSCV-IRES-GFP as previously described [37], [38]. The coding region of the resultant construct, designated as MSCV-huGPR4-IRES-GFP, was verified by DNA sequencing from both strands.

To generate the dominant-negative construct of Epac1 [39], a cDNA fragment encoding the N-terminal 420 amino acids of Epac1 was amplified by PCR from the HUVEC cDNA. The nucleotide sequence of the myc tag was designed to be added to the 5′ of the Epac1 sequence. A NotI site at the 5′ end and an EcoRI site at the 3′ end were designed for cloning into expression vectors. The PCR primers for amplifying the dominant-negative Epac1 fragment were: sense strand 5′- AGAGGGCGGCCGCACCATGGAGCAAAAGCTCATTTCTGAAGAGGACTTGGTGTTGAGAAGGATGCACCGGCCC -3′ and antisense strand 5′-GAGAGGAATTCCTACCTCTTGTTGCAGACGTAGGTGCT-3′. The amplified Epac fragment was digested with NotI and EcoRI and subcloned into the pQCXIP vector (Clontech, CA). The construct was sequenced to verify the accuracy of the coding sequence. The dominant-negative Epac construct was designated as pQCXIP-N-Epac.

### Site-directed mutagenesis

The DRY motif of GPCRs is important for G protein coupling and signaling [40]. A pair of primers was designed to generate an arginine to alanine mutation at the residue 115 (namely R115A) of the DRY motif of GPR4. The sequences of the primer pair were: sense strand 5′- TGCATCTCGGTGGACGCCTACCTGGCTGTG -3′ and antisense strand 5′- CACAGCCAGGTAGGCGTCCACCGAGATGCA -3′. The MSCV-huGPR4-IRES-GFP plasmid construct was used as the template and the R115A mutation was generated using the QuikChange site-directed mutagenesis kit (Stratagene, Inc., CA). The mutagenesis procedure was performed according to the manufacturer's instruction. The R115A mutation was confirmed by DNA sequencing and no other mutations were detected.

### RNAi knockdown

The microRNA-based short-hairpin RNA interference (miRNA) vector system (Invitrogen, Carlsbad, CA) was used for the RNAi knockdown of human GPR4 gene expression in HUVEC cells. Perfectly matched miRNA molecules mainly cause the degradation of target mRNAs. Several sets of miRNAs against human GPR4 were designed according to the manufacturer's instructions (www.invitrogen.com/rnai). The core nucleotide sequences of two effective human GPR4 miRNA molecules were: miRNA #1: (sense) 5′-ATCCCTCTACATCTTTGTCAT-3′ and (antisense) 5′-ATGACAAAGATGTAGAGGGAT-3′, and miRNA #2: (sense) 5′-CAAGAGGAACAGCACAGCCAA-3′ and (antisense) 5′-TTGGCTGTGCTGTTCCTCTTG-3′. A negative control miRNA that does not have sequence homology with any known genes was provided by Invitrogen. The core sequence of the negative control miRNA was: (sense) 5′-GTCTCCACGCGCAGTACATTT-3′ and (antisense) 5′-AAATGTACTGCGCGTGGAGAC-3′. The pre-miRNA oligos were cloned into the pcDNA 6.2-GW/RFP-miR vector and transferred to the lentiviral vector FCW attR1-attR2 using the BP/LR recombination reaction system (Invitrogen) as previously described [41]. All miRNA constructs were verified by DNA sequencing. The miRNA lentiviral vector was co-transfected with the packaging vectors pRSV-Rev, pCMV-VSV-G, and pMDL into HEK 293T cells (ATCC, Manassas, VA). Viral particles were produced and used to transduce HUVEC cells to express the miRNA molecules.

### Cell culture and retroviral transduction

All cells were cultured in a humidified tissue culture incubator with 5% CO_2_ and 95% air at 37°C. Primary HUVECs were purchased from Lonza (Walkersville, MD, previously known as Clonetics) and grown in endothelial cell growth medium 2 (EGM-2) (Lonza) as previously described [15]. U937 monocytic cell line was originally from American Type Culture Collection (ATCC, Manassas, VA). U937 cells were maintained in Dulbecco's modified Eagle's medium (DMEM) (Invitrogen) supplemented with 10% fetal bovine serum (FBS) (Invitrogen or HyClone, Logan, UT). To prepare retroviral particles for cell transduction, the MSCV or pQCXIP retroviral construct was co-transfected with the amphotropic packaging vector pCL-10A1 (Imgenex, CA) into HEK 293T cells (ATCC, Manassas, VA) to make replication-deficient viral particles, which were used to transduce HUVEC cells to express target genes. HUVECs stably expressing the MSCV-IRES-GFP, MSCV-huGPR4-IRES-GFP, or MSCV-huGPR4 R115A-IRES-GFP construct were isolated by fluorescence-activated cell sorting (FACS) based on green fluorescent protein (GFP) signals. HUVECs transduced with the pQCXIP or pQCXIP-N-Epac construct were selected with 2 µg/ml puromycin to enrich cells with the expression vector.

### Cell adhesion assay

The U937-HUVEC cell adhesion assay was performed as previously described [42], [43] with minor modifications. This approach examines the adhesion of U937 monocytic cells to a HUVEC monolayer under a static condition with no flow. Primary HUVECs from passage 3 to 7 were used for the cell adhesion assay. For isocapnic pH medium preparation, the pH buffering of cell culture media were carried out as previously described [15], [19]. Briefly, EGM-2 media were buffered with 7.5 mM HEPES, 7.5 mM EPPS and 7.5 mM MES (named as HEM), and the pH was adjusted using NaOH or HCl and measured with a pH meter (Fisher). To prepare hypercapnic pH media, EGM-2 media were added to cell culture plates and incubated in humidified tissue culture incubators filled with ambient air, 5% CO_2_ or 20% CO_2_, respectively. The pH of the EGM-2 media pre-treated under these conditions was measured to be around 8.4, 7.4 and 6.4, respectively. HUVECs stably expressing the MSCV-IRES-GFP vector, MSCV-huGPR4-IRES-GFP, MSCV-huGPR4 R115A-IRES-GFP, GPR4 miRNAs or control miRNA were cultured in a 24-well plate with EGM-2 media to form a confluent monolayer. For isocapnic pH treatment, HUVECs were incubated in the EGM-2/HEM media at various pHs for 5 h to 15 h (overnight) in a regular incubator with 5% CO_2_. For hypercapnic pH treatment, HUVECs were treated with CO_2_-buffered EGM-2 pH media for 5 h in incubators with ambient air, 5% CO_2_ or 20% CO_2_, respectively. After the pH treatment, HUVEC cells were washed once with DMEM at pH 7.4. To exclude the effects of acidosis on U937 monocytic cells, the step of U937 cell attachment to HUVEC cells was carried out at pH 7.4. U937 cells were resuspended in DMEM/HEM media with 10% FBS at pH 7.4, added at a density of 6×10^4^ cells/well in 1 ml of medium to a 24-well plate with HUVEC monolayer, and incubated for 1 h to allow cell attachment. Wells of the plate were gently washed with warm DMEM medium containing 10% FBS for at least 5 times to remove non-adherent U937 cells. Attached U937 cells from three random fields (excluding the edge of the wells which tended to have non-specific cell binding) were counted under an inverted microscope with a 10× objective (total 100× magnification) and the number of U937 cells per field was used as a functional readout to measure the adhesion capacity of HUVECs. To investigate the signaling pathways and adhesion molecules involved in acidosis/GPR4-induced cell adhesion, HUVECs were treated with various agents and blocking antibodies. The details of treatment conditions were described in the figure legends and/or in the results section.

### cAMP assay

The production of cAMP in HUVECs was measured using the Amersham cAMP Biotrak Enzymeimmunoassay (EIA) kit from GE Healthcare (Piscataway, NJ) following the manufacturer's instruction. Briefly, 2.5×10^4^ HUVEC cells/well were cultured in a 96-well plate and treated with 200 µl/well of EGM-2/HEM media or with CO_2_-buffered EGM-2 pH media at various pHs in the presence of 0.5 mM IBMX for 15 to 20 min. After the media were removed, 200 µl/well of lysis reagent 1B was immediately added to break cell membranes and release intracellular cAMP. 100 µl of cell lysate was transferred to a well of a donkey anti-rabbit Ig-coated plate. 100 µl of rabbit anti-cAMP antiserum was added and incubated for 2 h on ice, followed by the addition of 50 µl of cAMP-peroxidase conjugate and incubation for 1 h on ice. After all wells were washed with 400 µl of wash buffer four times, 150 µl of enzyme substrate was added into each well and incubated for 30 min at room temperature. The reaction was stopped by adding 100 µl/well of 1 M sulphuric acid, and the optical density was determined at 450 nm using a microplate reader (Molecular Devices, CA). The total intracellular cAMP was calculated according to the cAMP standard measured in parallel.

### Real-time RT-PCR (reverse transcriptase-polymerase chain reaction)

Human GPR4 gene expression was measured in HUVECs that were transduced with the control vector, GPR4 or miRNAs and grown in regular EGM-2 medium. The gene expression of endothelial adhesion molecules was assessed in HUVECs that were transduced with the control vector, GPR4 or GPR4 R115A mutant and treated with EGM-2/HEM media at pH 8.4, 7.4, and 6.4 or CO_2_-buffered EGM-2 pH media for 5 h. Total RNAs were extracted from HUVECs using the RNeasy Plus kit (QIAGEN, MD), and were reverse transcribed using the SuperScript II reverse transcriptase (Invitrogen, CA). Real-time PCR reagents were purchased from Applied Biosystems Inc (ABI, Foster City, CA). Primers specific for the adhesion molecules were the TaqMan Gene Expression Assays from ABI: VCAM-1, Hs01003372_ml; E-selectin, Hs00174057_ml; ICAM-1, Hs99999152_ml; P-selectin, Hs00174583_m1. The sequences of human GPR4 primers were: (sense) 5′-TTCGAGGAGCGCGTCTTT-3′, (antisense) 5′-GGTCCGCCACACAGTTGA-3′, and probe FAM-5′- CTGCATACCACAGCTCACTGGCTTTCAC -3′-TAMRA as previously described [44]. Human glyceraldehydes-3-phophate dehydrogenase (GAPDH) TaqMan Gene Expression Assay (ABI, Cat. # 4333764T) was used as the internal control. Real-time PCR was performed in duplicate with a program of 50°C for 2 min, 95°C for 10 min followed by 40 cycles of 95°C for 15 s and 60°C for 1 min, and the data was acquired and analyzed using the ABI 7300-HT real-time PCR thermocycler.

### Western blot analysis

HUVECs transduced with the myc-tagged N-Epac construct or the control pQCXIP vector were lysed in ice-cold RIPA lysis buffer (50 mM Tris-HCl, pH 7.4, 150 mM NaCl, 1% Triton X-100, 0.25% Na-deoxycholate, 1 mM EDTA, 1 mM phenylmethylsulfonyl fluoride, 1 mM Na_3_VO_4_, 1 mM NaF, 5% protease cocktail inhibitor from Sigma) for 15 min at 4°C. The cell lysates were clarified by centrifugation at 14,000× g for 15 min at 4°C. Protein concentration of the supernatant was determined by the Bradford protein assay (Bio-Rad, Hercules, CA). The expression of myc-tagged N-Epac was analyzed by Western blotting with the mouse monoclonal anti-c-myc antibody (Roche), followed by the incubation with the horseradish peroxidase (HRP)-conjugated goat anti-mouse IgG (H+L) secondary antibody (Bio-Rad). Chemiluminescence signals were detected using the Amersham ECL Advance Western blotting detection kit (GE Healthcare).

### Immunofluorescence

The immunostaining was performed as previously described with minor modifications [15], [45]. All staining steps were carried out at room temperature. Briefly, HUVEC/Vector and HUVEC/GPR4 cells were seeded in 8-well chamber slides, and grown overnight to allow cells to attach. Cells were then treated with EGM-2/HEM media at pH 8.4, 7.4, and 6.4 for 5 h, or with EGM-2 medium containing 10 nM thrombin for 20 min (for P-selectin only). After the treatment, cells were washed once with PBS and fixed with 4% paraformaldehyde in PBS for 15 min. Cells were then washed three times with PBS, permeabilized with PBST (PBS with 0.1% Tween 20) for 4 min and incubated with the blocking solution consisting of 5% normal goat serum in PBST for 30 min. For P-selectin staining, cells were fixed with 100% methanol at −20°C for 10 min, without the need for additional cell permeabilization. Afterwards, mouse monoclonal primary antibodies diluted in the blocking solution, including E-selectin antibody, VCAM-1 antibody, ICAM-1 antibody, or P-selectin antibody (R&D Systems), were applied to the cells at the concentration of 10 µg/ml and incubated for 1 h. After several washes with PBST, cells were incubated with Rhodamine Red-conjugated goat anti-mouse IgG secondary antibody (1∶200) (Invitrogen, R6393) for 1 h. Thereafter, unbound antibodies were removed by several washes with PBS, and cells were mounted in the anti-fade mounting medium (Vector Laboratories) and examined under a fluorescence microscope (Nikon) connected with a digital camera (Zeiss).

### Statistical analysis

Data were analyzed using the GraphPad Prism 5 software (GraphPad Software, Inc., CA) and depicted as mean ± SEM. *P*<0.05 was considered statistically significant.

## Results

### Activation of GPR4 by isocapnic acidosis increases HUVEC cell adhesion

**Figure 1:**
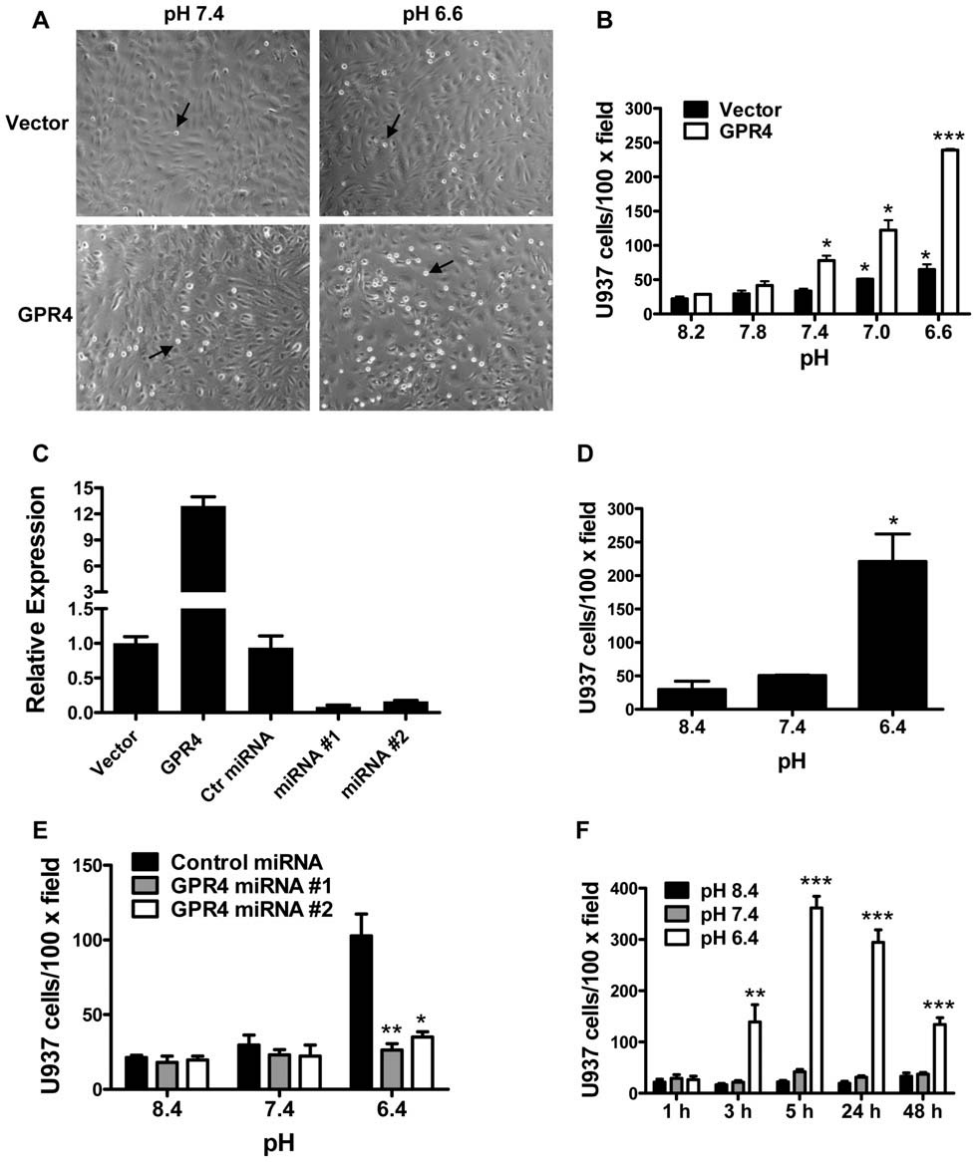
Acidosis/GPR4-induced adhesion of HUVEC cells. (A) HUVECs stably expressing the MSCV-huGPR4-IRES-GFP construct (GPR4) or the MSCV-IRES-GFP control vector (Vector) were grown as a flat monolayer and treated with EGM-2/HEM media at varying pH for either 5 h or 15 h (overnight) which showed similar results. Then, 6×10^4^ cells/well of U937 monocytic cells were added to adhere with HUVECs for 1 h at pH 7.4, and the static cell adhesion assay was performed as described in the “Materials and Methods”. After the adhesion assay, the attached U937 cells were readily detected as small, round and reflectile cells under an inverted microscope with a 10× objective. Arrows indicate attached U937 cells. (B) HUVECs stably overexpressing GPR4 or the control vector were treated with different pHs as indicated. The cell adhesion assay was then performed. *, *P*<0.05; ***, *P*<0.001; compared with the pH 8.2 group. (C) HUVECs were transduced with various genetic constructs as indicated. Total RNA was isolated and mRNA levels of GPR4 were determined by real-time RT-PCR. Values were normalized to the housekeeping gene GAPDH. The expression level of GPR4 in HUVECs with the control vector was set as 1. (D) HUVECs stably overexpressing the mouse GPR4-GFP fusion gene were treated with different pHs as indicated, and then the cell adhesion assay was performed. *Error bars* are the mean ± SEM. *, *P*<0.05, compared with pH 8.4. (E) HUVECs were transduced with two GPR4 miRNAs or control miRNA, followed by pH treatment, and then the cell adhesion assay was performed. *, *P*<0.05; **, *P*<0.01; compared with the control miRNA group at pH 6.4. (F) HUVEC/GPR4 cells were treated with different pHs for various lengths of time as indicated, and then the cell adhesion assay was performed. **, *P*<0.01; ***, *P*<0.001; compared with the 1 h group at pH 6.4. The results are depicted as the mean ± SEM and representative of more than two independent experiments.

Primary HUVECs, which have endogenous GPR4 expression, were used as a model system to study the effects of acidosis/GPR4 signaling on endothelial cell adhesion. HUVECs were stably transduced with the MSCV-IRES-GFP retroviral control vector (designated as HUVEC/Vector cells) or the MSCV-huGPR4-IRES-GFP construct (designated as HUVEC/GPR4 cells). To examine the adhesiveness of HUVEC cells and to model the interaction between endothelium and blood cells, we utilized the static HUVEC-U937 monocyte attachment assay as a functional readout [42], [43]. After non-adherent cells were removed by several washes, the U937 cells attached to the flat HUVEC monolayer could be readily detected as small, round and reflectile cells under an inverted microscope (Fig. 1A). For pH treatment, EGM-2 media were buffered with the chemicals HEPES, EPPS and MES (collectively as HEM) and the pH was adjusted with HCl or NaOH to generate isocapnic acidosis or alkalosis. When HUVEC/Vector cells were treated with pH 8.2, 7.8 and 7.4 (6.3 nM, 15.8 nM, and 40 nM H^+^, respectively), the cells exhibited a low capacity to adhere U937 monocytes. The treatment of pH 7.0 and 6.6 (100 nM and 251 nM H^+^, respectively) increased HUVEC/Vector cell adhesion by 2- to 4-fold (Fig. 1B). With the overexpression of human GPR4 in HUVEC/GPR4 cells, acidosis significantly augmented the adhesiveness of HUVEC cells by 5- to 10-fold (Fig. 1B). As measured by real-time RT-PCR, GPR4 was overexpressed about 12-fold in HUVEC/GPR4 cells when compared to HUVEC/Vector cells (Fig. 1C). Similarly, in HUVEC cells overexpressing the mouse GPR4-GFP fusion gene [15], [19], a significant increase of HUVEC adhesiveness was also observed upon acidic pH treatment (Fig. 1D). U937 cells cultured in RPMI medium showed similar results as that from the cells cultured in DMEM medium (Fig. S1A). In addition to U937 monocytes, the adhesion of HUVECs with another type of leukocytes, the HL-60 promyelocytic cells (neutrophils), under the static condition was also increased when HUVECs were stimulated by acidosis/GPR4 (Fig. S1B). These results suggest that activation of GPR4 by acidosis induces an adhesive phenotype of HUVECs.

To further investigate the role of GPR4 in endothelial cell adhesion induced by isocapnic acidosis, microRNAs-based short-hairpin RNA interference (miRNAs) was used to knockdown the endogenous GPR4 gene expression in HUVECs. We tested several miRNAs targeting different regions of GPR4 mRNA and identified two independent miRNAs that efficiently down-regulated GPR4 expression by more than 80% in HUVECs as measured by real-time RT-PCR (Fig. 1C). A negative control miRNA without sequence homology did not inhibit GPR4 expression. In the U937-HUVEC cell adhesion assay, acidosis increased the adhesiveness of HUVECs transduced with the negative control miRNA, but not HUVECs transduced with those two miRNAs that inhibited the endogenous GPR4 expression (Fig. 1E). These results suggest that the expression of GPR4 is required for acidosis-stimulated HUEVC adhesion.

We next examined the time course of acidosis/GPR4-induced endothelial cell adhesion. HUVEC/GPR4 cells were treated with EGM-2/HEM media at pH 6.4, 7.4 and 8.4 for 1, 3, 5, 24, and 48 hours and then U937 monocytes were added for the adhesion assay. As shown in Figure 1F, by 1 hour we did not observe an increase of HUVEC cell adhesion. By 3 hours, pH 6.4 (400 nM H^+^), compared to pH 8.4 (4 nM H^+^) and pH 7.4 (40 nM H^+^), significantly augmented the adhesiveness of HUVECs. By 5 to 24 hours, we observed the peak induction of HUVEC adhesion by acidic pH. By 48 hours, the induction was decreased when compared to that at 5 and 24 hours. The results suggest that the acidosis/GPR4-induced HUVEC adhesion occurs within 3 hours after the acidic pH stimulation.

### G_s_/cAMP signaling is important for acidosis/GPR4-induced HUVEC cell adhesion

**Figure 2:**
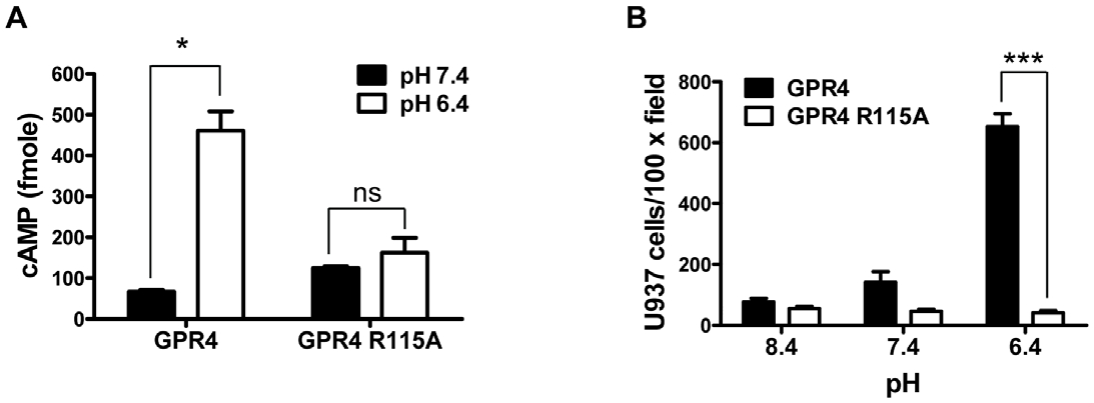
G protein signaling is indispensible for acidosis/GPR4-induced HUVEC adhesion. (A) HUVECs were transduced with GPR4 or the GPR4 R115A mutant expression vector. The intracellular cAMP level in HUVECs was determined by the cAMP assay as described under “Materials and Methods”. The total intracellular cAMP was calculated according to the cAMP standard measured in parallel. *, *P*<0.05; *ns*, not significant (*P*>0.05). (B) HUVEC cells as described in (A) were treated with indicated pHs and the cell adhesion assay was performed as described under “Materials and Methods”. ***, *P*<0.001. The results are representative of two independent experiments. *Error bars* are the mean ± SEM.

GPCRs are cell surface receptors that transduce signals mainly through G protein coupling [46], [47], [48], but G protein-independent pathways have also been reported [49]. Some GPCRs, particularly those with a long amino-terminal extracellular domain, can directly function as adhesion molecules to increase cell adhesion [50]. GPR4 does not have a long amino-terminal extracellular domain and thus is unlikely to serve as an adhesion molecule per se. Furthermore, we observed that the mRNA level of GPR4 was actually decreased by ∼50% in HUVEC/Vector cells upon acidosis treatment for 5 h (Fig. S2). This may represent a regulatory mechanism to down-regulate GPR4 signaling after its activation by acidosis, and the results also indicate that GPR4 itself may not directly act as an adhesion molecule to increase HUVEC adhesiveness. To elucidate GPR4 downstream pathways and to further rule out the possibility that GPR4 mediates cell adhesion by functioning as an adhesion molecule, we generated a GPR4 mutant with the arginine 115 to alanine (R115A) mutation in the DRY motif, which is located at the second intracellular loop and important for G protein signaling [38], [40]. The GPR4 R115A mutant was transduced into HUVEC cells (designated as HUVEC/GPR4 R115A cells) and the expression of the GPR4 R115A mutant was at the comparable level as that of GPR4 in HUVEC/GPR4 cells (data not shown). To examine the G protein signaling of the GPR4 R115A mutant, acidosis/GPR4-induced cAMP production was measured. Acidic pH stimulated the production of cAMP by about 7-fold in HUVEC/GPR4 cells but not in the HUVEC/GPR4 R115A cells (Fig. 2A), suggesting that the R115A mutant is defective in G protein signaling. Upon the treatment of pH 6.4, HUVECs overexpressing the wild-type GPR4 showed a ∼10-fold increase of adhesion with U937 cells. However, overexpression of the GPR4 R115A mutant did not augment HUVEC cell adhesion at acidic pH (Fig. 2B). These results indicate that G protein signaling is indispensable for acidosis/GPR4-mediated endothelial cell adhesion.

Since GPR4 was co-expressed with a bicistronic GFP marker, we examined whether the adhesiveness of HUVEC cells is linearly correlated with the intensity of GFP signal, an indicator of GPR4 expression. Overall, the GFP signal was heterogeneous and relatively low in the transduced HUVECs (Fig. S3). Some HUVEC cells with strong GFP signals did not always showed strong adhesion with U937 cells and, on the other hand, some HUVEC cells with low to medium GFP signals exhibited high adhesiveness (Fig. S3). The results suggest that acidosis/GPR4-induced HUVEC cell adhesion may not be linearly proportional to the overexpression of GPR4 per se but be related to downstream events triggered by GPR4 activation.

**Figure 3:**
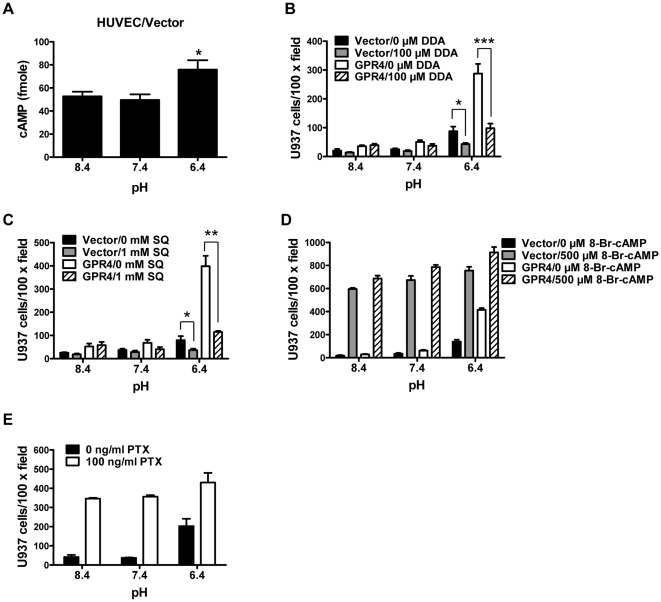
Involvement of cAMP in acidosis/GPR4-induced HUVEC adhesion. (A) The intracellular cAMP level in pH-treated HUVEC/Vector cells was determined by the cAMP assay as described in the “Materials and Methods”. *, *P*<0.05; compared with pH 8.4. (B) HUVECs stably overexpressing GPR4 or the control vector were pretreated with vehicle or 100 µM 2′,5′-dideoxyadenosine (DDA) for 1 h, followed by the treatment with indicated pH media containing vehicle or 100 µM DDA for 5 h, and the cell adhesion assay was performed as described in the “Materials and Methods”. *, *P*<0.05; ***, *P*<0.001. (C) HUVECs stably overexpressing GPR4 or the control vector were pretreated with vehicle or SQ 22536 (1 mM) for 1 h, followed by the treatment with indicated pH media containing vehicle or 1 mM SQ 22536 for 5 h, and then the cell adhesion assay was performed. *, *P*<0.05; **, *P*<0.01. (D) HUVECs stably overexpressing GPR4 or the control vector were treated with indicated pH media containing vehicle or 500 µM 8-bromo-cAMP for 15 h (overnight), and then the cell adhesion assay was performed. (E) HUVEC/GPR4 cells were treated with indicated pH media containing vehicle or 100 ng/ml PTX for 15 h, and then the cell adhesion assay was performed. All the above results are representative of at least two independent experiments. *Error bars* are the mean ± SEM.

To further delineate downstream pathways, we examined whether the G_s_/adenylate cyclase/cAMP pathway is important for acidosis/GPR4-induced endothelial cell adhesion. In addition to acidosis-induced cAMP production (∼7-fold) in HUVEC/GPR4 cells (Fig. 2A), a ∼1.5-fold increase of cAMP upon acidic pH stimulation was detected in HUVEC/Vector cells (Fig. 3A). Furthermore, inhibition of adenylate cyclase by 2′,5′-dideoxyadenosine (DDA) attenuated acidosis/GPR4-induced HUVEC adhesion (Fig. 3B). A similar extent of decrease in HUVEC adhesiveness was observed when cells were treated with another adenylate cyclase inhibitor SQ 22536 (Fig. 3C). Consistently, direct introduction of a cAMP analog, 8-bromo-cAMP, increased the adhesion of HUVECs at all tested pHs, recapitulating the effects of GPR4 activation (Fig. 3D). In line with this observation, treatment of HUVEC/GPR4 cells with pertussis toxin (PTX), which blocks the cAMP inhibitory G protein G_i_, increased the adhesion of HUVECs (Fig. 3E). These results indicate that the G_s_/cAMP pathway plays an important role in regulating acidosis/GPR4-mediated HUVEC adhesion.

### The cAMP effector, Epac, is involved in acidosis/GPR4-induced HUVEC cell adhesion

**Figure 4:**
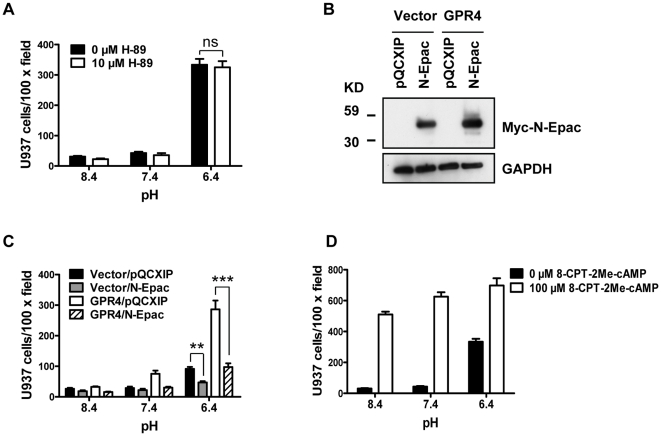
Epac is important for HUVEC adhesion induced by acidosis/GPR4. (A) HUVEC/GPR4 cells were pretreated with vehicle or H-89 (10 µM) for 1 h. Cells were then treated with indicated pH media containing vehicle or 10 µM H-89 for 5 to 15 h (overnight), and the cell adhesion assay was performed as described under “Materials and Methods”. *ns*, not significant (*P*>0.05). (B) HUVEC/Vector or HUVEC/GPR4 cells were stably transduced with the myc-tagged Epac dominant-negative construct N-Epac or the control vector pQCXIP. Total proteins were extracted from HUVEC cells and subject to Western blot analysis for myc-N-Epac with anti-myc antibodies. GAPDH was used as a loading control. (C) HUVEC/Vector or HUVEC/GPR4 cells were stably transduced with the dominant-negative N-Epac or the control vector pQCXIP. Cells were treated with indicated pH media for 5 or 15 h and then the cell adhesion assay was performed. **, *P*<0.01; ***, *P*<0.001. (D) HUVEC/GPR4 cells were treated with indicated pH media containing vehicle or 100 µM 8-CPT-2Me-cAMP for 15 h, and then the cell adhesion assay was performed. All the above results are representative of two or more independent experiments. *Error bars* are the mean ± SEM.

Increase of intracellular cAMP can activate several downstream effectors including protein kinase A (PKA) and Epac [51]. To determine which effector(s) are involved in acidosis/GPR4-induced HUVEC adhesion, we first examined the role of the best characterized cAMP target, PKA, in this process. Treating HUVEC/GPR4 cells with the PKA inhibitor H-89 did not affect cell adhesion induced by acidosis (Fig. 4A), suggesting that PKA is not important for this process. To verify that H-89 effectively inhibited the activity of PKA, we examined the phosphorylation status of the PKA substrate CREB (cAMP response element-binding) by Western blotting and showed that H-89 effectively blunted CREB phosphorylation induced by 8-bromo-cAMP in HUVECs (data not shown). Next, to elucidate the role of Epac in acidosis/GPR4-mediated HUVEC adhesion, a dominant-negative Epac mutant N-Epac or the pQCXIP control vector was expressed in HUVEC/Vector and HUVEC/GPR4 cells. The N-Epac dominant-negative mutant lacks the kinase domain and blocks the activity of Epac by competitively binding with the substrates [39]. By Western blotting with a myc antibody, the exogenous expression of myc-N-Epac was confirmed in HUVEC/Vector and HUVEC/GPR4 cells (Fig. 4B). In the HUVEC-U937 adhesion assay, the expression of the N-Epac mutant inhibited acidosis/GPR4-induced HUVEC cell adhesion (Fig. 4C). On the other hand, when HUVEC cells were directly treated with the Epac specific activator 8-CPT-2Me-cAMP [52], cell adhesion was enhanced at all pHs tested (Fig. 4D). These results suggest that Epac, but not PKA, is the downstream cAMP effector that mediates acidosis/GPR4-induced HUVEC adhesion.

### Activation of GPR4 by acidosis increases the expression of endothelial adhesion molecules E-selectin, VCAM-1, and ICAM-1

**Figure 5:**
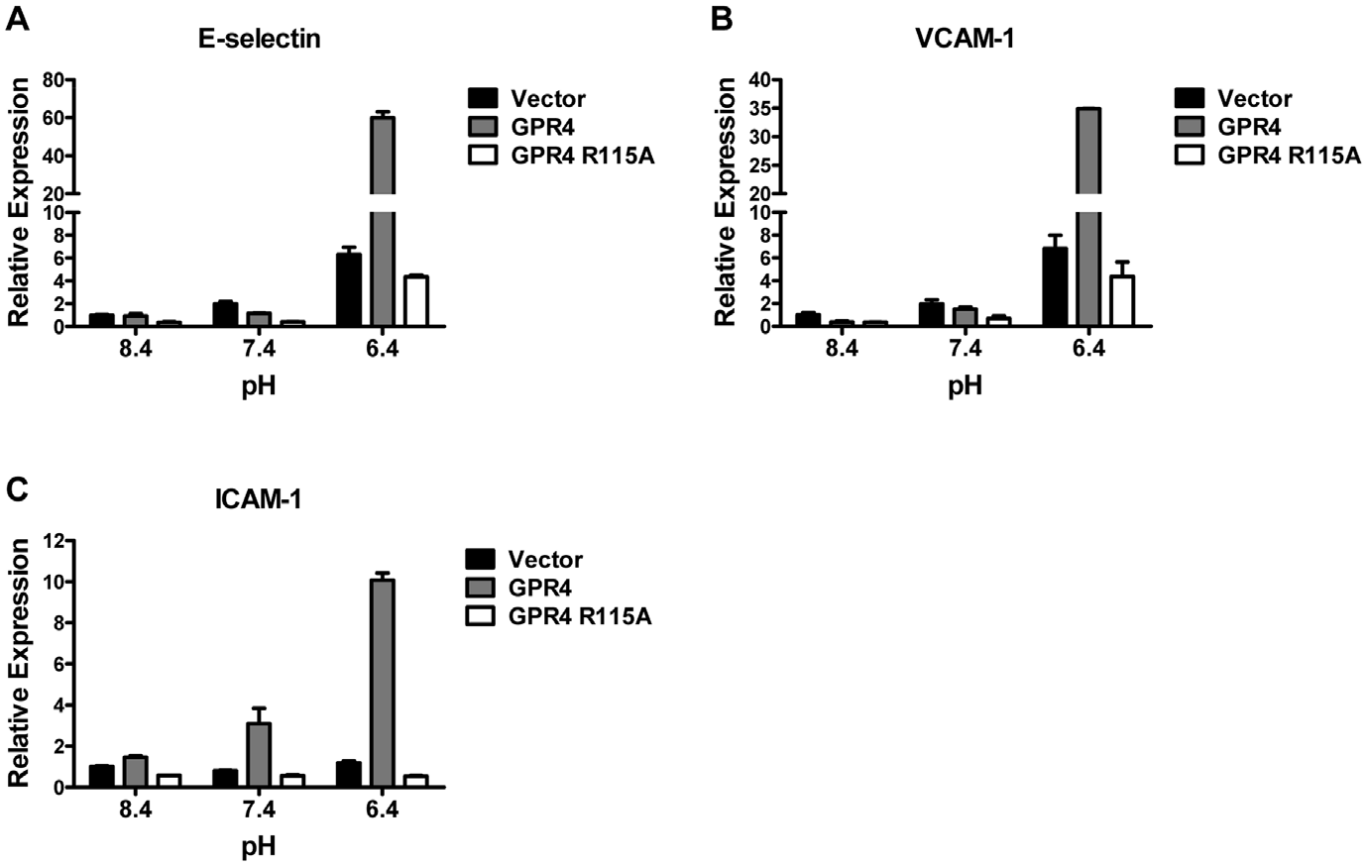
Real-time RT-PCR of adhesion molecules E-selectin, VCAM-1, and ICAM-1. HUVECs transduced with the control vector, GPR4 or GPR4 R115A mutant were treated with EGM-2/HEM media at pH 8.4, 7.4, or 6.4 for 5 h. Total RNAs were isolated and reverse transcribed. Real-time RT-PCR quantification of mRNA levels of E-selectin (A), VCAM-1 (B), and ICAM-1 (C) was performed in duplicate. Values were normalized to the housekeeping gene GAPDH. The expression level of the target gene in HUVEC/Vector cells at pH 8.4 was set as 1. The results are representative of at least two independent experiments. *Error bars* are the mean ± SEM.

During leukocyte extravasation, the early steps like leukocyte tethering, rolling and firm adhesion are associated with elevated expression of endothelial cell adhesion molecules, such as E-selectin, vascular cell adhesion molecule-1 (VCAM-1), and intercellular adhesion molecule-1 (ICAM-1), three extensively characterized endothelial adhesion molecules [53], [54]. To investigate the gene expression levels of these adhesion molecules, we used real-time RT-PCR to examine their mRNA levels in pH-treated HUVEC cells transduced with different GPR4 constructs. Levels of VCAM-1 and E-selectin mRNAs were increased by about 7-fold upon acidosis treatment in HUVEC/Vector cells expressing the endogenous level of GPR4. Moreover, overexpression of GPR4 in synergy with acidosis further enhanced the gene expression of VCAM-1 and E-selectin substantially (Figs. 5A and B). Acidosis/GPR4 stimulated the expression of ICAM-1, however, to a lesser extent as there was both a minor increase in HUVEC/Vector cells and an increase of 10-fold in HUVEC/GPR4 cells (Fig. 5C). In contrast, overexpression of the GPR4 R115A mutant failed to further augment the mRNA levels of these adhesion molecules upon acidosis stimulation in HUVEC cells (Figs. 5A, B and C). These results are in accordance with the U937 monocyte adhesion assay results (Figs. 1B and 2B).

**Figure 6:**
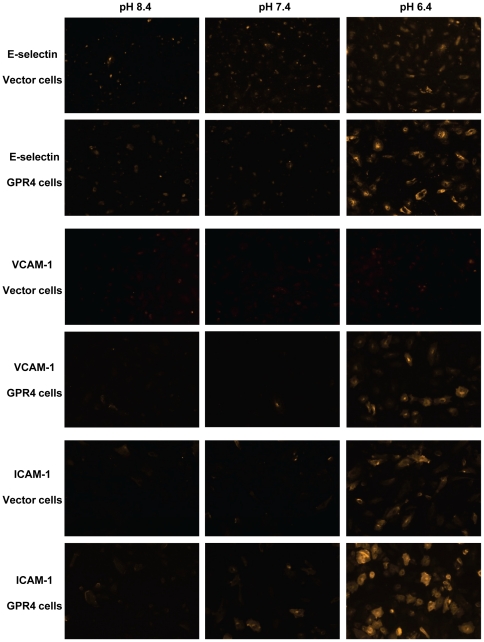
Immunofluorescence staining of E-selectin, VCAM-1 and ICAM-1 in HUVEC cells. After treated with EGM-2/HEM media at pH 8.4, 7.4, or 6.4 for 5 h, HUVEC/Vector and HUVEC/GPR4 cells were fixed with 4% paraformaldehyde, incubated with E-selectin, VCAM-1 or ICAM-1 primary antibody, Rhodamine Red-conjugated secondary antibody, and then detected under a fluorescence microscope (10× objective). For an accurate comparison of fluorescence signals, each group of images (e.g. E-selectin/Vectors cells, pH 8.4, pH 7.4, and pH 6.4) was taken with the same exposure time. The results are representative of three independent experiments.

Immunofluorescence staining was performed to assess the expression of E-selectin, VCAM-1, and ICAM-1 at the protein level. When HUVEC/Vector and HUVEC/GPR4 cells were treated with pH 8.4 and pH 7.4, the expression of E-selectin, VCAM-1, and ICAM-1 was at the basal level. Acidic pH 6.4 treatment stimulated the expression of E-selectin and ICAM-1 in HUVEC/Vector cells and the expression was further increased in HUVEC/GPR4 cells (Fig. 6). While VCAM-1 was barely detectable by this antibody in HUVEC/Vector cells, its expression was clearly up-regulated by pH 6.4 in HUVEC/GPR4 cells (Fig. 6). We also examined whether acidosis/GPR4 could induce the expression or membrane translocation of P-selectin, another adhesion molecule in endothelial cells [55], [56], [57]. The real-time RT-PCR result did not show any significant up-regulation of P-selectin mRNA in HUVECs induced by acidosis/GPR4 (Fig. S4A). Moreover, we could not detect cell membrane translocation of P-selectin protein from the Weibel-Palade body by the acidosis treatment for 5 h (Fig. S4B) or 20 min (data not shown). In comparison, the thrombin treatment for 20 min as a positive control induced the translocation of P-selectin to cell membrane (Fig. S4B).

Collectively, the results suggest that activation of GPR4 by acidosis in HUVEC cells increases the expression of multiple endothelial adhesion molecules including E-selectin, VCAM-1 and ICAM-1, which may interact with their receptors on U937 cells to mediate the adhesion.

### Blockade of endothelial E-selectin, VCAM-1, and ICAM-1 on HUVECs reduces acidosis/GPR4-mediated cell adhesion

**Figure 7:**
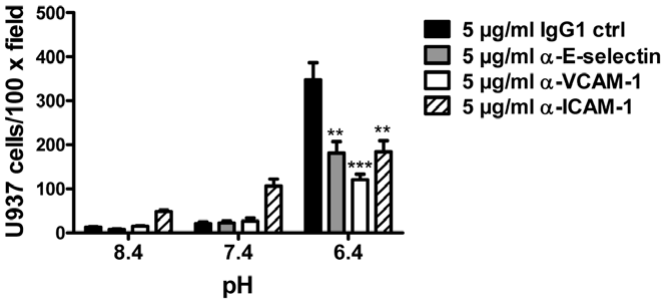
Blockade of E-selectin, VCAM-1 or ICAM-1 reduces the adhesion of HUVECs with U937 monocytic cells. HUVECs stably overexpressing GPR4 were treated with different pHs as indicated for 5 to 15 h, followed by 30 min incubation with pH 7.4 medium containing 5 µg/ml IgG1 control antibody, anti-E-selectin, anti-VCAM-1, or anti-ICAM-1 antibody. The cell adhesion assay was then performed as described under “Materials and Methods”. **, *P*<0.01; ***, *P*<0.001; compared with the IgG1 control group at pH 6.4. The results are representative of two independent experiments. *Error bars* are the mean ± SEM.

Monoclonal blocking antibodies were used to examine whether E-selectin, VCAM-1, and ICAM-1 are functionally involved in acidosis/GPR4-induced HUVEC adhesion. After the treatment with different pHs for 5 h, HUVEC/GPR4 cells were incubated with E-selectin, VCAM-1, ICAM-1 blocking antibodies or the mouse IgG1 control and the static cell adhesion assay was performed. Compared to the IgG1 control, each of the three blocking antibodies significantly decreased the adhesion of HUVECs with U937 monocytes (Fig. 7). It was also noted that the ICAM-1 antibody caused an increase of basal adhesion for unknown reasons. The blocking antibody results suggest that multiple adhesion molecules, including E-selectin, VCAM-1 and ICAM-1, are functionally important for acidosis/GPR4-induced HUVEC adhesion with U937 monocytes.

### Hypercapnic acidosis can also activate GPR4 to increase the adhesion molecule expression and the adhesiveness of HUVECs

**Figure 8:**
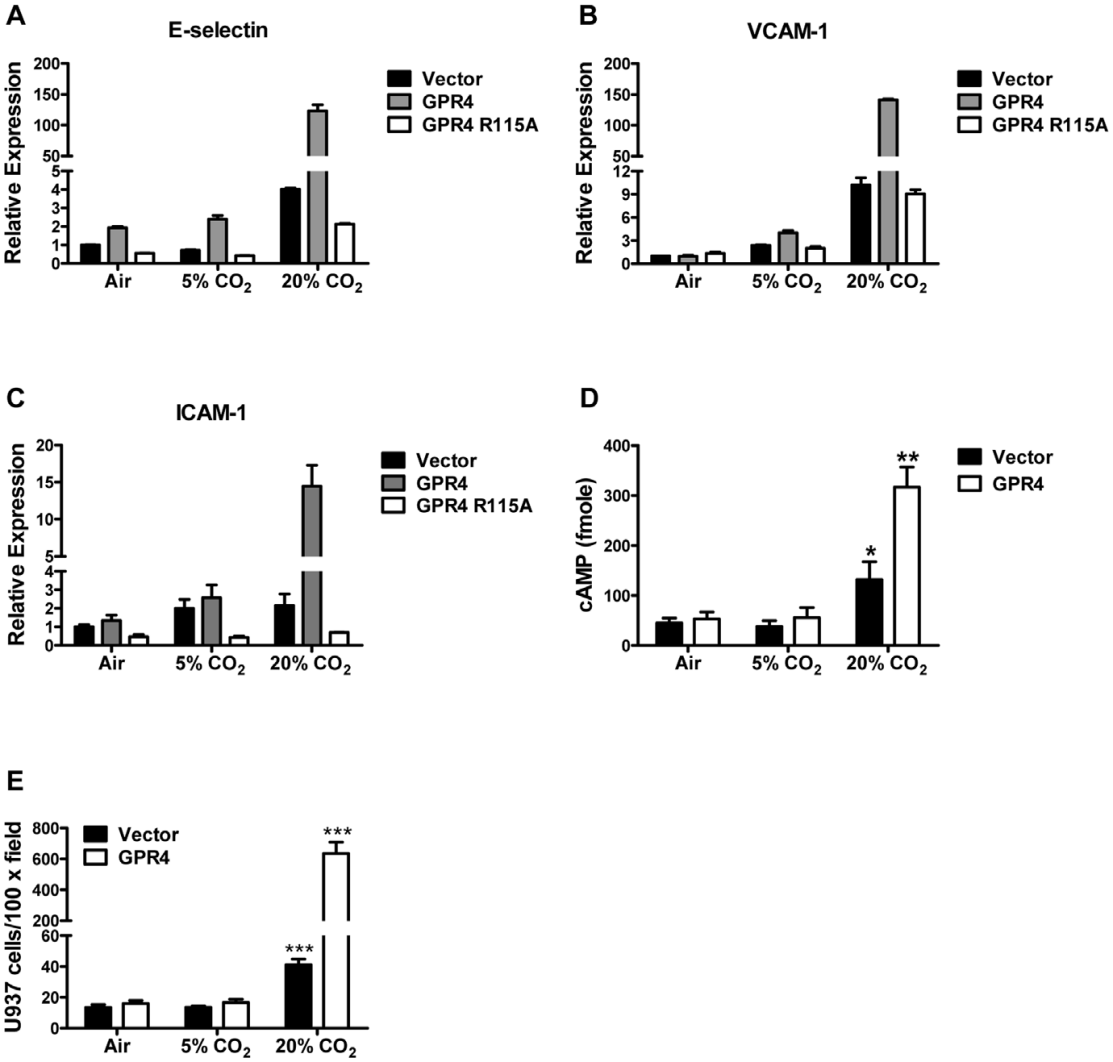
Activation of GPR4 by hypercapnic acidosis stimulates HUVEC adhesion molecule expression, cAMP production, and adhesiveness. (A–C) HUVEC/Vector, HUVEC/GPR4, or HUVEC/GPR4 R115A cells were treated with ambient air, 5% CO_2_, or 20% CO_2_-buffered EGM-2 pH media for 5 h. Total RNA was isolated and mRNA levels of E-selectin (A), VCAM-1 (B), and ICAM-1 (C) were determined by real-time RT-PCR. Values were normalized to the housekeeping gene GAPDH. The expression level of the target gene in HUVEC/Vector cells under ambient air (∼pH 8.4) was set as 1. (D) The intracellular cAMP level in HUVEC/Vector and HUVEC/GPR4 cells treated with the CO_2_-buffered pH media was determined by the cAMP assay as described in the “Materials and Methods”. *, *P*<0.05; **, *P*<0.01; compared with the 5% CO_2_ group. (E) HUVEC/Vector and HUVEC/GPR4 cells were treated with the CO_2_-buffered pH media as indicated for 5 h. The cell adhesion assay was performed as described in the “Materials and Methods”. ***, *P*<0.001; compared with the 5% CO_2_ group. All the above results are representative of at least two independent experiments. *Error bars* are the mean ± SEM.

In all the results described above, HUVECs were treated with isocapnic acidosis in which EGM-2 media were buffered with HEPES, EPPS and MES and the acidic pH was obtained by adding HCl. Hypercapnic acidosis, caused by an increase in carbon dioxide in tissues, blood or body fluid, is another type of acidosis that is associated with many pathological conditions [9], [11]. To investigate the effects of hypercapnic acidosis on GPR4 signaling, HUVEC/Vector and HUVEC/GPR4 cells were treated with EGM-2 culture media under ambient air (∼0.04% CO_2_), 5% CO_2_ and 20% CO_2_. The pHs of the EGM-2 media under these levels of CO_2_ were around 8.4, 7.4 and 6.4, respectively. As measured by real-time RT-PCR, the hypercapnic acidosis treatment (20% CO_2_), in comparison to 5% CO_2_ and ambient air, increased the expression of E-selectin, VCAM-1 and ICAM-1 mRNA by several fold in HUVEC/Vector cells, and the expression was further substantially induced in HUVEC/GPR4 cells but not HUVEC/GPR4 R115A mutant cells (Figs. 8A, B and C). Moreover, the hypercapnic acidosis (20% CO_2_) treatment stimulated the production of cAMP in HUVEC/Vector cells, with further increase in HUVEC/GPR4 cells (Fig. 8D). Concordantly, the hypercapnic treatment augmented the adhesiveness of HUVEC/Vector cells, and this effect was particularly robust in HUVEC/GPR4 cells (Fig. 8E). These results suggest that, similar as isocapnic acidosis, hypercapnic acidosis can also activate GPR4 to induce the expression of the endothelial adhesion molecules, stimulate cAMP production, and increase HUVEC adhesiveness.

## Discussion

Acidosis has been shown to regulate vascular tone, blood vessel growth, and the function of smooth muscle and endothelial cells [12], [58], [59]. At the molecular level, ATP-sensitive potassium channels, activated by intracellular acidic pH in vascular smooth muscle cells, are involved in acidosis-induced coronary arteriolar dilation [60], [61]. Recent studies indicate that extracellular acidosis activates acid-sensing ion channels (ASIC) to regulate the function of smooth muscle cells [62]. We have recently shown that extracellular acidosis activates GPR4 in endothelial cells and leads to cAMP production and the inhibition of endothelial cell migration and microvessel growth [15], suggesting that GPR4 is a functional pH sensor in endothelial cells.

In this report, we have identified a novel function of the GPR4 receptor. Activation of GPR4 by acidosis enhances HUVEC adhesiveness and binding with U937 monocytes. This effect is augmented by GPR4 overexpression and diminished by GPR4 knockdown in HUVECs. Since the interaction between endothelial cells and leukocytes is critical for inflammatory response, acidosis/GPR4 signaling may represent a new pathway in inflammation. With particular relevance, local interstitial acidosis is commonly found in inflammatory tissues. For instance, acidic tissue pH is associated with asthma, arthritis, and cystic fibrosis [2], [4], [6]. Acidosis is also frequently identified in ischemia and solid tumors in which inflammation plays an important role in disease progression. Interestingly, it has been shown that inflammatory stimuli TNF-α and H_2_O_2_ can induce GPR4 mRNA expression in human brain microvascular endothelial cells but not dermal microvascular endothelial cells [63]. These data suggest that the acidosis/GPR4 pathway may serve as a potential target for modulating vascular adhesion, endothelium-blood cell interaction, and inflammatory responses.

Acidosis can modulate the expression of a wide range of genes in various cell systems such as renal, intestinal, and cancer cells [64], [65], [66]. Previous studies showed that acidosis is important for hypoxia/inflammatory stimuli-induced ICAM-1 expression in cultured endothelial cells [67]. Acidification of extracellular pH increases the expression of a key inflammatory enzyme cyclooxygenase-2 (COX-2) in endothelial cells [68]. However, the pH-sensing mechanisms of endothelial cells in response to acidosis are not well defined. Our results suggest that GPR4 is a novel sensor for vascular endothelial cells to respond to acidic tissue microenvironments and elicit inflammatory responses. Data presented here reveal that acidosis activates GPR4 to increase endothelial cell adhesiveness through multiple adhesion molecules such as E-selectin, VCAM-1, and ICAM-1, which then facilitate the binding of leukocytes.

We have also shown that hypercapnic acidosis, similar as isocapnic acidosis, can activate GPR4 to increase the expression of adhesion molecules and the adhesive capacity of HUVECs. In the literature, there are seemingly conflicted reports regarding the effects of hypercapnia on endothelial cell adhesion. Takeshita et al. showed that hypercapnia decreased the lipopolysaccharide (LPS)-induced upregulation of ICAM-1 in human pulmonary artery endothelial cells [69]. However, Liu et al. recently demonstrated that hypercapnia increased the LPS-induced expression of inflammatory molecules such as ICAM-1, VCAM-1 and E-selectin in human pulmonary microvascular endothelial cells and in a LPS-induced lung injury animal model [70]. Our own results using HUVECs as a model system are in line with the observations by Liu et al. [70] but in contrast to the reports by Takeshita et al. [69]. Although the reasons for the paradoxical findings are not exactly clear, the use of endothelial cells from different vessel types has been proposed as a potential explanation [70]. Indeed, endothelial cells isolated from different types of blood vessels have been shown to exhibit different phenotypes, gene expression profiles and responses to stimuli [71], [72]. It should also be noted that hypercapnia leads to the increase of not only protons but also carbon dioxide and bicarbonate, all of which can trigger downstream signaling pathways through different mechanisms [11]. Thus, hypercapnia may elicit more complicated biological effects than hypercapnic acidosis alone. In the HUVEC model system, our results strongly suggest that hypercapnic acidosis can activate the proton-sensing receptor GPR4 in a gene dose-dependent manner. This observation may have implications in certain hypercapnia-associated clinical problems. For instance, the low-tidal-volume mechanical ventilation, used for the treatment of acute lung injury (ALI) and acute respiratory distress syndrome (ARDS), commonly causes hypercapnia [73]. While some studies indicate that hypercapnia is potentially beneficial in attenuating lung inflammation during ventilation, other studies suggest the opposite [9], [69], [70], [74], [75]. These observations underscore the complex biological effects of hypercapnia and the importance of further research to better understand the manifold pro- and anti-inflammatory signaling pathways related to hypercapnia. In this regard, GPR4 signaling may represent a novel pathway that senses hypercapnic acidosis and modulates inflammatory responses.

Moreover, we have further demonstrated that G protein signaling and the G_s_/adenylate cyclase/cAMP/Epac pathway are important for acidosis/GPR4-induced HUVEC adhesion. This conclusion is supported by several lines of evidence. First, the R115A DRY motif mutant of GPR4, which is deficient in G protein signaling, fails to increase HUVEC adhesiveness in response to acidosis. Second, inhibition of adenylate cyclase or Epac substantially blunts the acidosis/GPR4-induced HUVEC adhesion. Third, treatment with the cAMP analog 8-bromo-cAMP or the Epac specific activator 8-CPT-2Me-cAMP recapitulates the HUVEC adhesive phenotype induced by acidosis/GPR4 signaling. Our observations are in line with several previous studies. Rangarajan et al. demonstrate that upon the stimulation of β2-adrenergic receptor, cAMP induces the adhesion of Ovcar3 ovarian cancer cells through the Epac/Rap1 pathway [34]. Fukuhara et al. show that the cAMP/Epac pathway increases VE-cadherin-mediated cell-cell contact and promotes endothelial barrier function [76]. Our results indicate that the G_s_/cAMP/Epac pathway is also involved in acidosis/GPR4-induced endothelial cell adhesion, illustrating a recurrent theme in signaling transduction that diverse stimuli and receptor activation may converge into common downstream signaling pathways. It should also be pointed out that activation of GPR4 by acidosis has been shown to stimulate multiple G protein pathways including G_s_, G_q_ and G_13_, of which the physiological role of G_q_ and G_13_ pathways in endothelial cell acidosis responses is not clear and needs to be further studied.

Emerging evidence suggests that the GPR4 family of proton-sensing GPCRs, including GPR4, TDAG8, OGR1 and G2A, plays a role in inflammation. Previous studies have demonstrated that acidosis activates TDAG8 to produce cAMP in thymocytes and splenocytes [19], [77]. Stimulation of TDAG8 by acidosis regulates the viability of eosinophils and the production of cytokines [78], [79]. Our results show that activation of GPR4 by acidosis increases the adhesiveness of endothelial cells. While we have not studied the effects of acidosis/GPCR signaling on leukocyte adhesiveness, this aspect warrants further investigation because the reciprocal interaction between endothelial cells and leukocytes is critical for inflammation. Current data indicate that the proton-sensing GPCRs, such as GPR4 and TDAG8, potentially serve as the pH sensors for vascular cells and blood cells to perceive the acidic microenvironment at inflammatory sites and transduce signaling pathways to regulate the function of blood vessels and leukocytes. As acidosis is closely associated with inflammation, ischemia, cancer, and other diseases, the GPR4 proton-sensing receptor family may play a role in these pathological conditions.

## Supporting Information

Figure S1 **Acidosis/GPR4-induced HUVEC adhesion using different types of culture media or leukocytes.** (A) HUVEC/Vector and HUVEC/GPR4 cells were treated with EGM-2/HEM media at indicated pHs for 5 to 15 h. RPMI medium supplemented with 10% FBS was used to replace DMEM medium for growing U937 cells and washing the plate to remove non-adhered U937 cells. The cell adhesion assay was performed as described in the “Materials and Methods”. **, *P*<0.01; compared with the pH 8.4 group. (B) HUVEC/Vector and HUVEC/GPR4 cells were treated with EGM-2/HEM media at different pHs as indicated for 5 to 15 h. Afterwards, 6×10^4^ cells/well of HL-60 promyelocytic cells were added to adhere with HUVECs for 1 h at pH 7.4, and the cell adhesion assay was performed. **, *P*<0.01; ***, *P*<0.001; compared with the pH 8.4 group. The results are representative of two or more independent experiments. *Error bars* are the mean ± SEM.
(TIF)

Figure S2 **Endogenous mRNA level of GPR4 in HUVECs is decreased during acidic pH treatment.** HUVEC/Vector cells were treated with EGM-2/HEM media at pH 8.4, 7.4 or 6.4 pH for 5 h. Total RNAs were isolated and the level of GPR4 mRNA was determined by real-time RT-PCR. Values were normalized to the housekeeping gene GAPDH. The expression level of GPR4 at pH 8.4 was set as 1. *Error bars* are the mean ± SEM. ***, *P*<0.001; compared with pH 8.4.
(TIF)

Figure S3 **Correlation between the intensity of GFP signal and the adhesiveness of HUVEC cells.** In HUVEC/GPR4 cells, GPR4 was co-expressed with a bicistronic GFP marker, which can serve as an indicator of GPR4 expression. HUVEC/GPR4 cells were treated with EGM-2/HEM medium at pH 6.4 for 5 h. The cell adhesion assay was performed as described under “Materials and Methods”. After the adhesion assay, HUVEC/GPR4 cells and attached U937 cells were detected under an inverted fluorescence microscope (Zeiss) with a 10× objective. Micrographs of phase contrast (A) or GFP signal (B) of cells in the same field were taken and compared. In the phase contrast picture (A), the large and flat cells were HUVECs and the small, round and reflectile cells were attached U937 cells. Arrows indicate the areas with U937 cell attachment in the phase contrast picture (A) and the corresponding GFP signal of HUVECs in the fluorescence picture (B).
(TIF)

Figure S4 **P-selectin mRNA expression and protein translocation are not affected by acidosis/GPR4 in HUVECs.** (A) HUVEC/Vector, HUVEC/GPR4, and HUVEC/GPR4 R115A cells were treated with EGM-2/HEM media at pH 8.4, 7.4 or 6.4 for 5 h. Total RNA was isolated and mRNA levels of P-selectin were determined by real-time RT-PCR. Values were normalized to the housekeeping gene GAPDH. The expression level of P-selectin at pH 8.4 was set as 1. The results are representative of two independent experiments. *Error bars* are the mean ± SEM. (B) HUVEC/Vector and HUVEC/GPR4 cells were treated with EGM-2/HEM media at pH 8.4, 7.4, or 6.4 for 5 h, or with EGM-2 medium containing 10 nM thrombin for 20 min. After the treatment, cells were fixed with 100% methanol, incubated with P-selectin primary antibody, Rhodamine Red-conjugated secondary antibody, and then detected under a fluorescence microscope (100× objective). Thrombin treatment served as the positive control for P-selectin translocation. Weibel-Palade (WP) bodies that contain P-selectin are shown as bright particles indicated by short arrows. The translocation of WP bodies from cytoplasm to cell membrane is indicated by solid arrows in the thrombin treatment groups. The results are representative of three independent experiments.
(TIF)
