# Supplementary figures and images for: Activation of GPR4 by Acidosis Increases Endothelial Cell Adhesion through the cAMP/Epac Pathway
Source: PLoS One. 2011 Nov 16;6(11):e27586. doi: 10.1371/journal.pone.0027586 (PMC3217975; doi:10.1371/journal.pone.0027586)

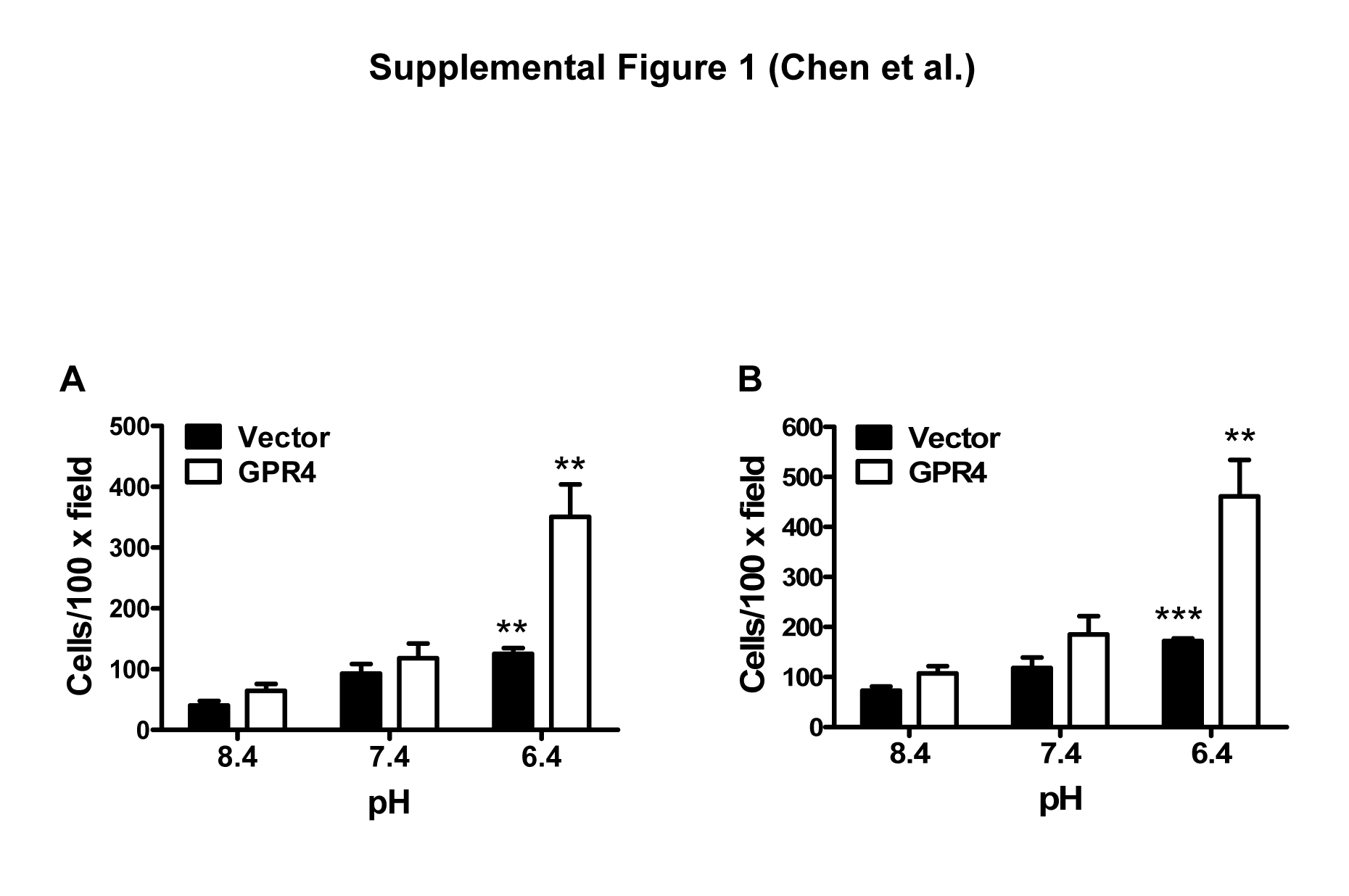

Supplement: Figure S1 — Acidosis/GPR4-induced HUVEC adhesion using different types of culture media or leukocytes. (A) HUVEC/Vector and HUVEC/GPR4 cells were treated with EGM-2/HEM media at indicated pHs for 5 to 15 h. RPMI medium supplemented with 10% FBS was used to replace DMEM medium for growing U937 cells and washing the plate to remove non-adhered U937 cells. The cell adhesion assay was performed as described in the “Materials and Methods”. **, P<0.01; compared with the pH 8.4 group. (B) HUVEC/Vector and HUVEC/GPR4 cells were treated with EGM-2/HEM media at different pHs as indicated for 5 to 15 h. Afterwards, 6×10^4^ cells/well of HL-60 promyelocytic cells were added to adhere with HUVECs for 1 h at pH 7.4, and the cell adhesion assay was performed. **, P<0.01; ***, P<0.001; compared with the pH 8.4 group. The results are representative of two or more independent experiments. Error bars are the mean ± SEM. (TIF) [file pone.0027586.s001.tif]

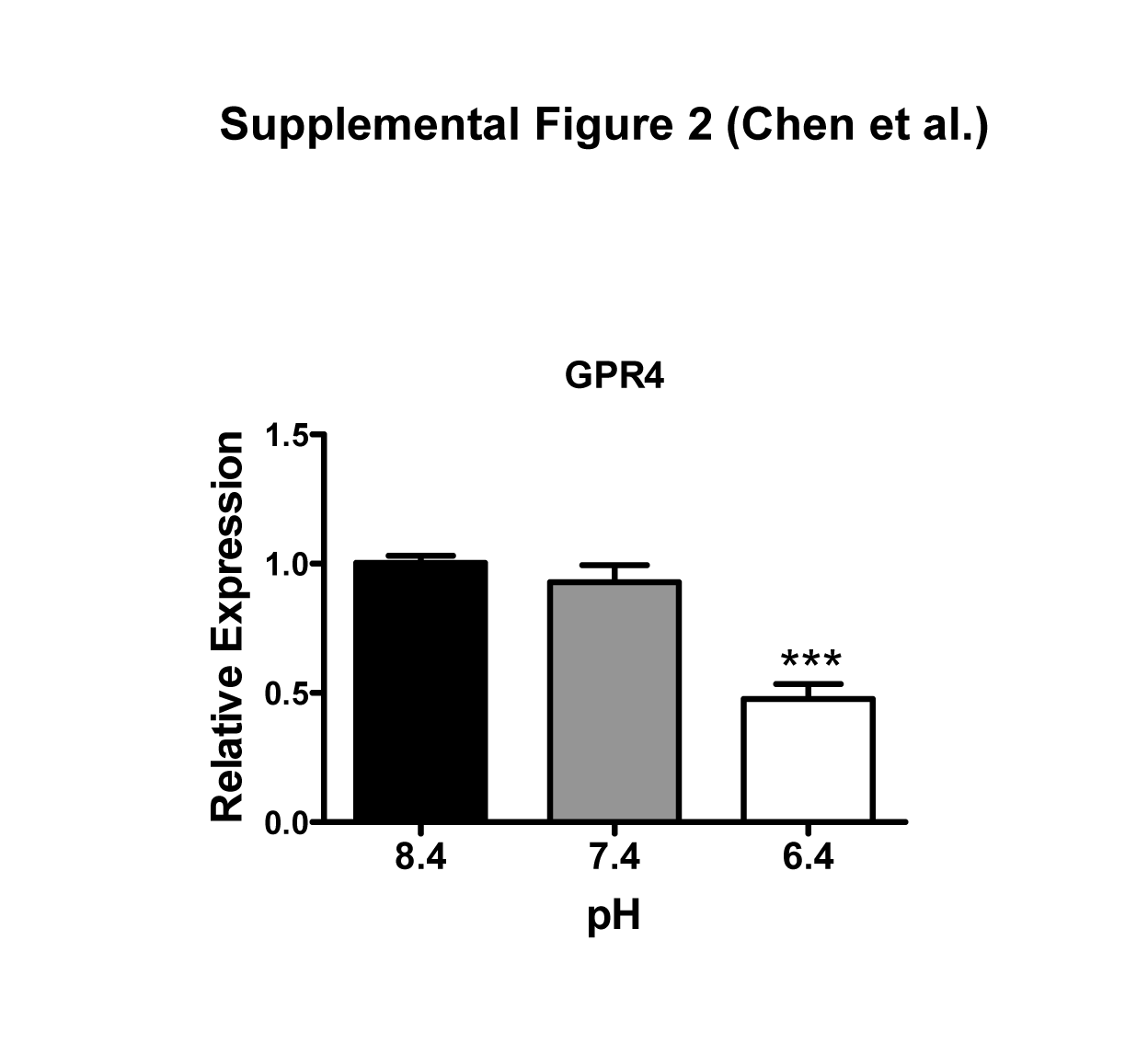

Supplement: Figure S2 — Endogenous mRNA level of GPR4 in HUVECs is decreased during acidic pH treatment. HUVEC/Vector cells were treated with EGM-2/HEM media at pH 8.4, 7.4 or 6.4 pH for 5 h. Total RNAs were isolated and the level of GPR4 mRNA was determined by real-time RT-PCR. Values were normalized to the housekeeping gene GAPDH. The expression level of GPR4 at pH 8.4 was set as 1. Error bars are the mean ± SEM. ***, P<0.001; compared with pH 8.4. (TIF) [file pone.0027586.s002.tif]

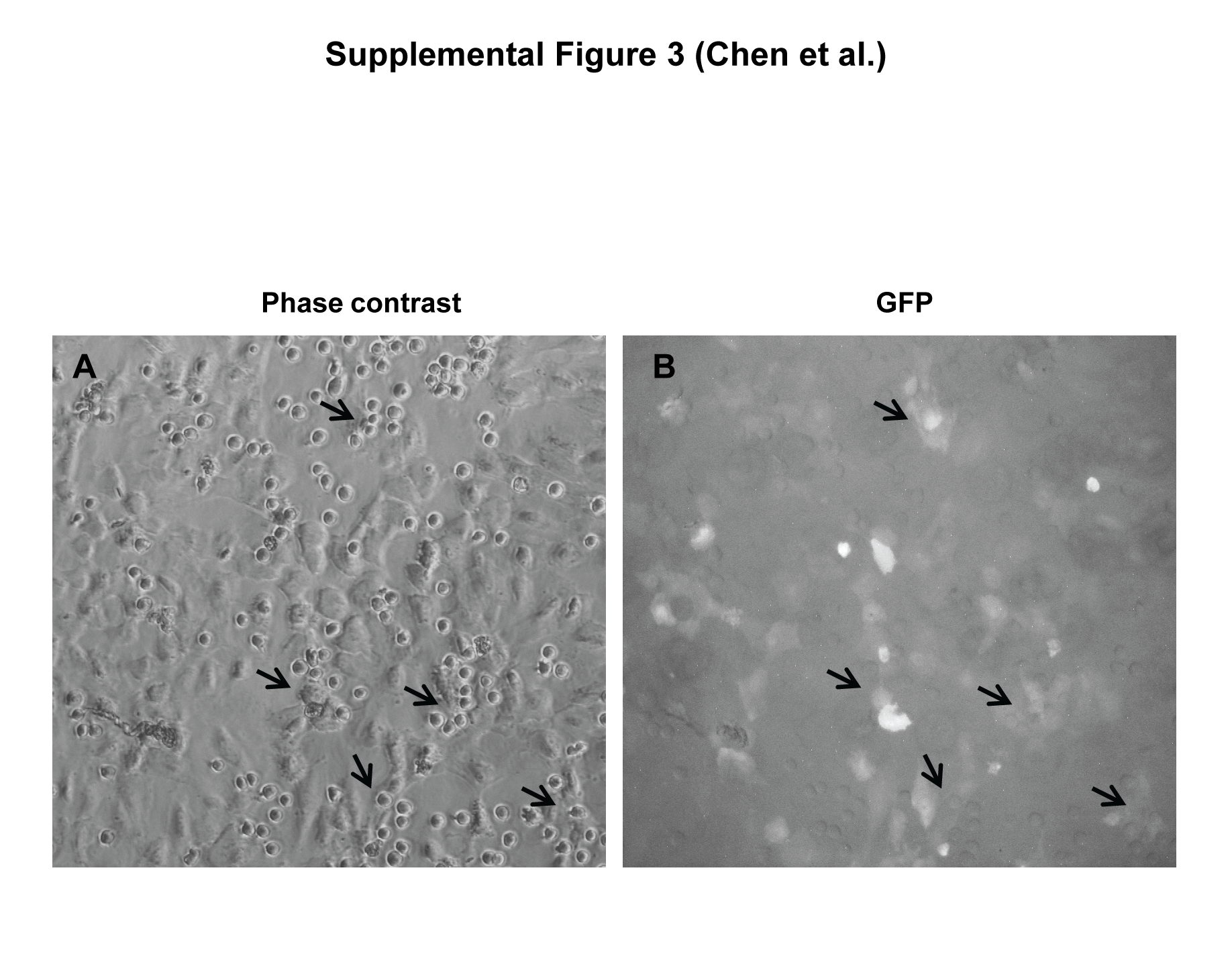

Supplement: Figure S3 — Correlation between the intensity of GFP signal and the adhesiveness of HUVEC cells. In HUVEC/GPR4 cells, GPR4 was co-expressed with a bicistronic GFP marker, which can serve as an indicator of GPR4 expression. HUVEC/GPR4 cells were treated with EGM-2/HEM medium at pH 6.4 for 5 h. The cell adhesion assay was performed as described under “Materials and Methods”. After the adhesion assay, HUVEC/GPR4 cells and attached U937 cells were detected under an inverted fluorescence microscope (Zeiss) with a 10× objective. Micrographs of phase contrast (A) or GFP signal (B) of cells in the same field were taken and compared. In the phase contrast picture (A), the large and flat cells were HUVECs and the small, round and reflectile cells were attached U937 cells. Arrows indicate the areas with U937 cell attachment in the phase contrast picture (A) and the corresponding GFP signal of HUVECs in the fluorescence picture (B). (TIF) [file pone.0027586.s003.tif]

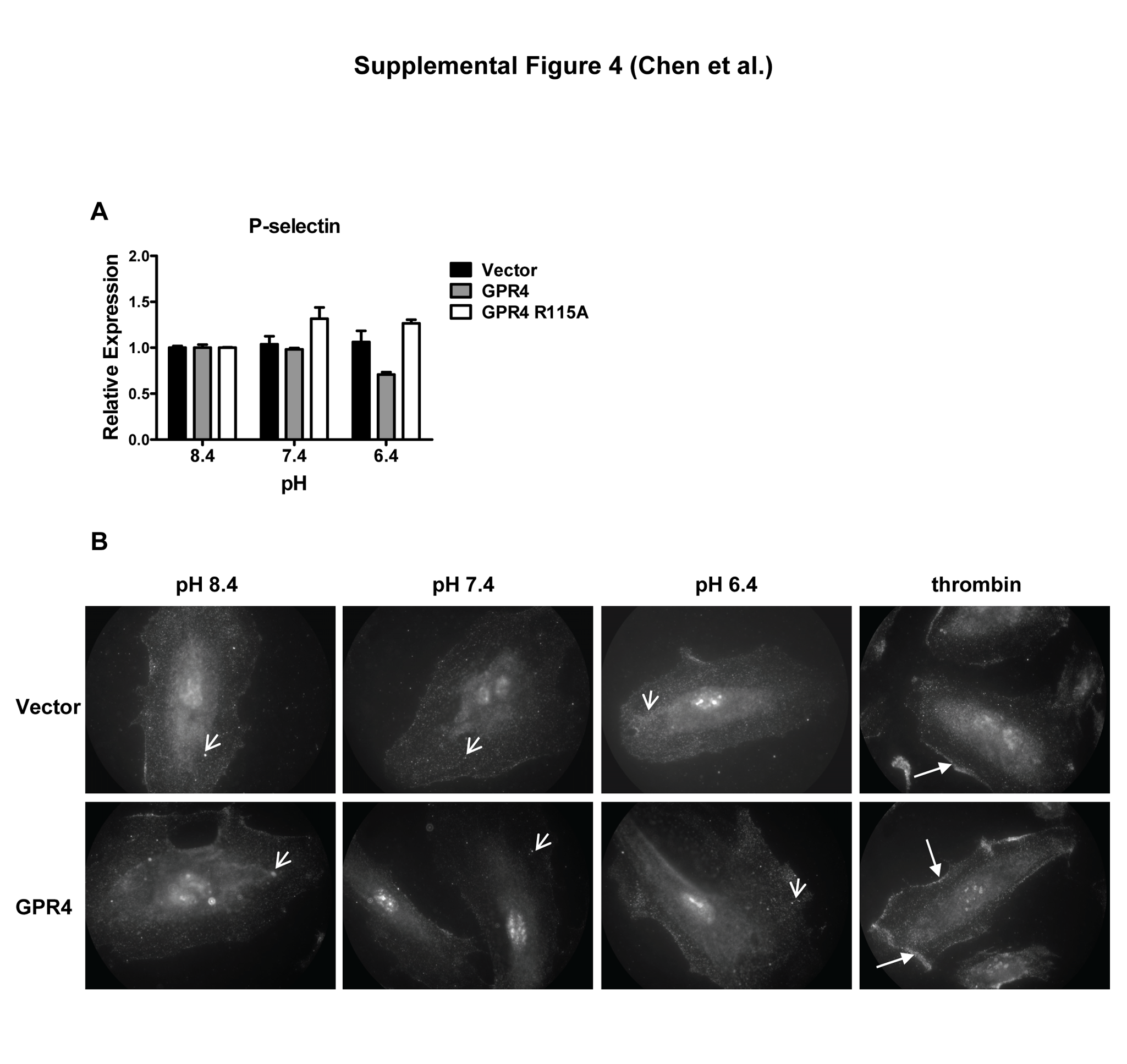

Supplement: Figure S4 — P-selectin mRNA expression and protein translocation are not affected by acidosis/GPR4 in HUVECs. (A) HUVEC/Vector, HUVEC/GPR4, and HUVEC/GPR4 R115A cells were treated with EGM-2/HEM media at pH 8.4, 7.4 or 6.4 for 5 h. Total RNA was isolated and mRNA levels of P-selectin were determined by real-time RT-PCR. Values were normalized to the housekeeping gene GAPDH. The expression level of P-selectin at pH 8.4 was set as 1. The results are representative of two independent experiments. Error bars are the mean ± SEM. (B) HUVEC/Vector and HUVEC/GPR4 cells were treated with EGM-2/HEM media at pH 8.4, 7.4, or 6.4 for 5 h, or with EGM-2 medium containing 10 nM thrombin for 20 min. After the treatment, cells were fixed with 100% methanol, incubated with P-selectin primary antibody, Rhodamine Red-conjugated secondary antibody, and then detected under a fluorescence microscope (100× objective). Thrombin treatment served as the positive control for P-selectin translocation. Weibel-Palade (WP) bodies that contain P-selectin are shown as bright particles indicated by short arrows. The translocation of WP bodies from cytoplasm to cell membrane is indicated by solid arrows in the thrombin treatment groups. The results are representative of three independent experiments. (TIF) [file pone.0027586.s004.tif]
